# Implementation of a Clinical Decision Support Alert for the Management of *Clostridium difficile* Infection

**DOI:** 10.3390/antibiotics4040667

**Published:** 2015-12-21

**Authors:** Sara Revolinski

**Affiliations:** Froedtert & the Medical College of Wisconsin, 9200 W Wisconsin Avenue, Milwaukee, WI 53226, USA; E-Mail: sara.revolinski@froedtert.com; Tel.: +1-414-805-2678

**Keywords:** clinical decision support, *Clostridium difficile*, order set

## Abstract

*Clostridium difficile* infections are common in hospitalized patients and can result in significant morbidity and mortality. It is imperative to optimize the management of *C. difficile* infections to help minimize disease complications. Antimicrobial stewardship techniques including guidelines, order sets and other clinical decision support functionalities may be utilized to assist with therapy optimization. We implemented a novel alert within our electronic medical record to direct providers to the *C. difficile* order set in order to assist with initiating therapy consistent with institutional guideline recommendations. The alert succeeded in significantly increasing order set utilization, but guideline compliance was unchanged.

## 1. Introduction

*Clostridium difficile* is currently the most common cause of nosocomial infections in the United States, with parallel increases also being reported in the community [1]. In 2011, *C. difficle* was reported to have caused 453,000 infections and 29,000 deaths [1]. *C. difficile* is also associated with significant morbidity, including treatment failure, recurrent disease, increased hospitalizations and longer hospital lengths of stay [2,3,4,5].

In order to minimize disease complications, there is a need to ensure that therapies for *C. difficile* are optimized. The Infectious Diseases Society of America (IDSA) and the American College of Gastroenterology (ACG) both currently maintain practice guidelines for the management of *Clostridium difficile* infections (CDIs), which outline recommended treatment regimens [6,7]. These guidelines suggest the use of metronidazole for mild to moderate CDIs, vancomycin for severe, and the combination of intravenous (IV) metronidazole and vancomycin for complicated diseases [6,7].

Antimicrobial stewardship programs are often involved with the management of CDIs. One component of stewardship is to ensure that patients are receiving optimal care for their infections. Stewardship programs can utilize several methods to ensure appropriate treatment, including guideline development and antimicrobial order forms [8]. Previous studies have shown when order sets are utilized, compliance with guidelines, dosing and core measures is increased, but minimal studies have evaluated methods to increase order set utilization [9,10].

While guidelines typically outline the most current evidence-based practice, these recommendations may be difficult to apply in practice for a variety of reasons. With the abundance of information in healthcare today, it is impossible for every provider to remain current with all of the literature [11]. There is a need to assist providers in rational prescribing, and clinical decision support is one avenue that may allow for this [11]. Order sets built within a system’s electronic medical record (EMR) can assist with guideline compliance, but utilization of order sets varies between institutions and providers [8]. For institutions where order sets are not commonly utilized, other clinical decision support modalities must be trialed. This article describes one such modality and its impact on the care of patients affected by CDIs.

## 2. Experimental Section

### 2.1. Study Design

This single-center, quasi-experimental study evaluated patients with a CDI before and after the implementation of a *C. difficile* best practice alert (BPA). The BPA was implemented on 9 February 2015, and was an additional clinical decision support tool layered over existing functionality within our EMR. Adult inpatients (aged ≥18 years) were included if they had a nucleic acid amplification test (NAAT) result positive for *C. difficile* along with provider-determined symptoms of *C. difficile* colitis between 1 October 2014 and 8 February 2015 (historical group) or 9 February 2015 and 31 May 2015 (intervention group). Patients were excluded if they were pregnant, initiated on antibiotic therapy for prophylaxis of *C. difficile*, deemed to be colonized with *C. difficile* without active colitis, or received therapy for CDIs immediately prior to admission. No other interventions for the management of CDIs were implemented during the study period. 

### 2.2. Institutional Clinical Decision Support for C. difficile

**Figure 1:**
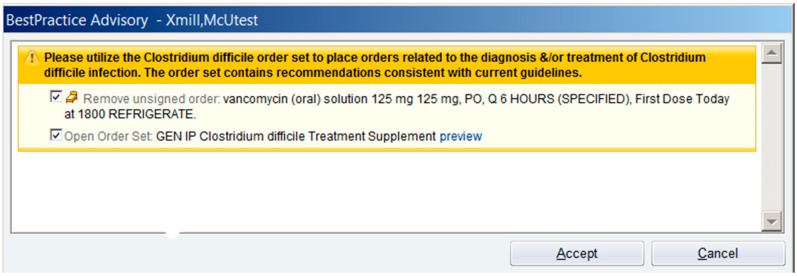
*Clostridium difficile* Best Practice Alert. © 2015 Epic Systems Corporation. Used with permission.

Our institution implemented an order set for CDIs to align with our institutional guideline for the treatment and management of *C. difficile* in early 2013. This order set contained recommendations for the treatment of mild to moderate, severe, severe-complicated, and recurrent infections that mirrored the recommendations from the IDSA [6]. Mild to moderate CDIs were defined as serum creatinine (Scr) less than 1.5 times the patient’s baseline and a white blood cell (WBC) count less than 15,000 cells/microliter; patients with mild to moderate CDIs were recommended to receive metronidazole. Patients meeting one or both of the above criteria (Scr at least 1.5 times baseline or WBC 15,000 cells/microliter or greater) were considered to have severe diseases and were recommended vancomycin. Complicated diseases included patients with ileus, toxic megacolon, bowel perforation, sepsis, hypotension, and renal dysfunction. Patients with complicated diseases were recommended metronidazole IV in combination with oral vancomycin, and the use of a vancomycin enema was also considered. First recurrences were treated similarly, however patients with 2 or more recurrences were recommended a full course of vancomycin that was subsequently tapered. In April 2014, our institution began to track order set utilization and found it to be minimal, with one use recognized over the first 6 months of implementation. This underutilization prompted the implementation of additional clinical decision support functionality in the form of a BPA. The BPA was activated upon order entry for either a *C. difficile* NAAT or oral vancomycin, and offered the clinician the opportunity to utilize the *C. difficile* order set to ensure selected therapy was consistent with guideline recommended therapy (Figure 1). When the provider chose to accept the alert, he was taken directly to the order set where he could then place orders for the management of the CDI, at which point the initial order he entered was discontinued. The provider was also able to cancel the BPA and continue with the original order that was entered. To avoid alert fatigue, it was decided that this alert would not be activated when an order was placed for oral or intravenous metronidazole, as this antibiotic is often used to treat infections other than *C. difficile.*

### 2.3. Outcomes

The goal of this study was to examine the effect of additional clinical decision support functionality on the management of *C. difficile* infections at our institution. The primary objective was to evaluate overall guideline compliance for the treatment of CDIs. Secondary objectives included describing the utilization rate of the *C. difficile* order set and determining the impact the order set had on guideline compliance. 

### 2.4. Statistical Analysis

Demographic data were analyzed using descriptive statistics with dichotomous data analyzed using Fisher’s exact test and continuous data using the 2-tailed Student *t* test. A *p*-value ≤ 0.05 was considered statistically significant. 

## 3. Results and Discussion 

### 3.1. Results

**Figure 2:**
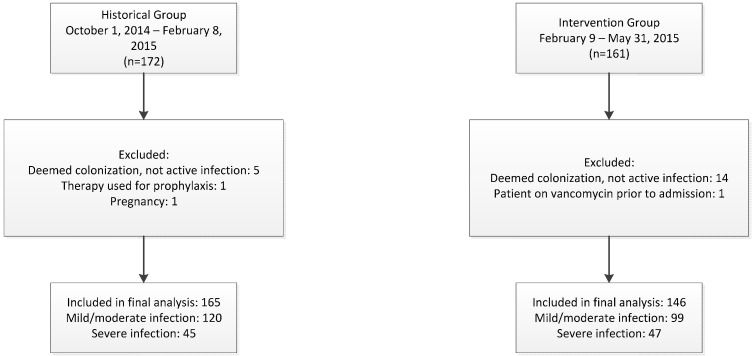
Study participants.

A total of 333 patients were admitted to the hospital with a positive *C. difficile* NAAT result during the entire study period, with 311 patients included in the final analysis. One hundred sixty-five patients were included in the historical group and 146 in the intervention group (Figure 2). Demographics were similar between groups with the exception of intensive care unit (ICU) admissions upon positive NAAT result, with significantly more patients in the intervention group admitted to the ICU, and patients in the historical group displaying significantly lower albumin levels (Table 1). 

**Table 1:** Demographics.

Demographics	Historical Group (n = 165)	Intervention Group (n = 146)	*p* Value
Age, y, mean ± SD	60.7 ± 17.3	58.6 ± 14.9	0.184
Male sex, no. (%)	79 (47.9)	69 (47.3)	0.292
BMI, mean ± SD	29.5 ± 10.1	29.3 ± 8.7	0.970
Albumin, mean ± SD	3.2 ± 0.8	3.4 ± 0.7	0.029
Mild/moderate infection, no. (%)	120 (72.7)	99 (67.8)	0.384
Severe infection, no. (%)	45 (27.3)	47 (32.2)	0.384
Complicated infection, no (%)	25 (15.2)	29 (19.9)	0.296
ICU admission when NAAT positive, no. (%)	29 (17.6)	46 (31.5)	0.005
Hospital-acquired, no. (%)	66 (40.0)	70 (47.9)	0.176
Infectious diseases consult, no. (%)	22 (13.3)	21 (14.4)	0.870
Patients with recurrent CDIs, no. (%)	34 (20.6)	41 (28.1)	0.144

Only two (1.2%) patients were prescribed antibiotics for CDIs directly from the order set during the historical period, compared to 45 (30.8%) in the intervention period (*p* < 0.0001) (Table 2). Compliance with the guideline was similar before and after implementation of the BPA despite a significant increase in *C. difficile* order set utilization. After the implementation of the BPA, significantly more patients received metronidazole for a severe infection (vancomycin is the antibiotic of choice in this situation), and significantly fewer patients with severe complicated infections did not receive combination therapy; however, when the patients in the intervention group were stratified by the use of the order set, these differences did not remain significant (Table 2).

**Table 2:** Guideline Compliance and Order Set Utilization.

	Historical Group n = 165	Intervention Group n = 146	*p* Value	Intervention Group With Use of Order Set n = 45	Intervention Group Without Use of Order Set n = 101	*p* Value
Order set utilization	2 (1.2)	45 (30.8)	<0.001	NA	NA	NA
Treatment compliant with guideline	115 (69.7)	104 (71.2)	0.605	36 (80.0)	68 (67.3)	0.165
Reasons for Non-Compliance						
Given metronidazole for severe infection	10/50 (20.0)	20/45 * (44.4)	0.015	4/9 (44.4)	16/36 * (44.4)	1.000
Given vancomycin for mild/moderate infection	12/50 (24.0)	5/45 (11.1)	0.116	1/9 (11.1)	4/36 (11.1)	1.000
Combination therapy given but not needed	6/50 (12.0)	7/45 (15.6)	0.767	3/9 (33.3)	4/36 (11.1)	0.131
Patient required combination therapy but did not receive	17/50 (34.0)	4/45 (8.9)	0.006	0/9 (0.0)	4/36 (11.1)	0.569
Vancomycin taper indicated but not given	2/50 (4.0)	4/45 (8.9)	0.418	1/9 (11.1)	3/36 (8.3)	1.000
Other **	3/50 (6.0)	5/45 (11.1)	0.470	0/9 (0.0)	5/36 (13.9)	0.566

All data presented as number (%); * Some patients had multiple reasons for noncompliance with guideline; ** Other reasons included incorrect dosing or route of antibiotics, allergy to preferred agent, drug interaction with preferred agent, previously treated with alternative agent.

In the intervention group, there were 48 (32.9%) patients who were initially prescribed vancomycin. The BPA was triggered by all of these patients and was bypassed 18 times, resulting in the order set being utilized in 30 patients initially prescribed vancomycin. Consequently, guideline compliance was evaluated in patients who initially received vancomycin and was compared to those patients who did not initially receive vancomycin (Table 3), since patients receiving vancomycin were more likely to receive the BPA.

**Table 3:** Order Set Utilization and Guideline Compliance Based on Initial Antibiotic Therapy.

	Initial Treatment Regimen Included Vancomycin n = 48	Initial Treatment Regimen Did Not Include Vancomycin n = 98	*p* value
Order set utilization, no. (%)	30/48 (62.5)	15/98 (15.3)	<0.001
Treatment compliant with guideline	29/48 (60.4)	71/98 (74.4)	0.184
Guideline compliance when order set utilized	22/30 (73.3)	11/15 (73.3)	1.000
Guideline compliance when order set not utilized	7/18 (38.9)	60/83 (72.3)	0.012

### 3.2. Discussion

CDIs are associated with high morbidity and mortality, and it is thus imperative to initiate proper evidence-based treatment to minimize adverse outcomes. The management of *C. difficile* is not straightforward and can vary based on the severity of the infection and the number of infection recurrences. Because of this, clinical decision support outlining the best prescribing practice would be beneficial to providers. Order sets can steer providers toward evidence-based prescribing, but are not always utilized within an EMR. The *C. difficile* BPA at our institution was developed to increase the utilization of the *C. difficile* order set, thereby intending to increase compliance with evidence-based treatment recommendations. To our knowledge, there are no other articles supporting the utilization of this type of alert to promote order set utilization and guideline compliance. 

The BPA was successful with increasing utilization of the *C. difficile* order set, but utilization of the order set remained low overall. Only 30.8% of patients with CDIs benefitted from order set utilization post-implementation. Despite the increase in order set utilization, guideline compliance remained unchanged. It was encouraging, however, to see that the order set was employed in 15 patients in the intervention group where the BPA was not triggered, suggesting that the BPA has increased awareness of the presence of the order set. The utilization of order sets is recommended as a supplemental strategy for antimicrobial stewardship to guide optimal antimicrobial prescribing [8]. Unfortunately, in our institution, increasing access to the order set did not correlate with optimized prescribing, suggesting that passive mechanisms of stewardship interventions are minimally successful. 

There were several limitations to this study. One limitation is the low overall utilization of the order set despite implementation of the BPA. The reasons for alert activation likely contributed to this, as the majority of patients in this study were categorized as having mild to moderate infections. At the time of this study, mild to moderate infections were treated with metronidazole at our institution, and metronidazole does not trigger the BPA alert. While forcing the alert to activate for metronidazole orders may have increased order set utilization, it would have also come at the risk of alert fatigue due to the unnecessary activating for patients without *C. difficile* infection.

Additionally, the retrospective nature of this study limited the assessment of the severity of the infections in these patients. It is possible that therapy choices were made based on clinical presentation and purposely deviated from the guideline. Moreover, the definition of mild to moderate and severe diseases utilized in our internal guideline and the IDSA guideline is limited, since it only takes into account acute renal dysfunction and leukocytosis [6]. These criteria are difficult to assess in select patient populations, such as those with chronic kidney disease, those receiving dialysis, and patients with neutropenia. The criteria do not account for age, albumin status, or frequency of bowel movements—criteria that have been suggested in the literature to predict disease severity [7,12]. It is also possible that providers preferentially select vancomycin for patients with mild to severe CDIs based on the results of literature published after the release of the IDSA and ACG guidelines [13].

While the implementation of an alert to promote order set usage is a novel mechanism not previously documented in the literature, this study suggests that passive interventions may not be successful in our institution. Beginning in November 2015, our institution will be implementing a *C. difficile* treatment bundle that will be employed by clinical pharmacists upon result of a positive *C. difficile* NAAT. Part of this bundle will require pharmacists to ensure that treatment for CDIs are in line with the recommendations in our guideline and to intervene if there is discordance. Also of note, our guideline has been updated since this study to no longer include metronidazole as a recommended treatment option for mild to moderate CDIs (vancomycin is recommended for mild to severe infection). If, after the implementation of this bundle, we find that the order set is still not guiding providers to the correct antibiotics, the order set will be redesigned or the BPA will be retired. 

## 4. Conclusions

Additional clinical decision support through use of a BPA to help guide antimicrobial prescribing for CDIs was minimally successful at our institution. The BPA did increase the use of the order set for CDIs, however this did not result in increased rates of guideline-concordant prescribing. Passive efforts to encourage prescribing that reflects our internal guideline may be unsuccessful at our institution. 
